# Analysis of Avian Influenza (H5N5) Viruses Isolated in the Southwestern European Part of the Russian Federation in 2020–2021

**DOI:** 10.3390/v14122725

**Published:** 2022-12-06

**Authors:** Nikolay Zinyakov, Artem Andriyasov, Pavel Zhestkov, Anton Kozlov, Zoya Nikonova, Evgeniya Ovchinnikova, Alena Grekhneva, Lidiya Shcherbakova, Dmitriy Andreychuk, Alexander Sprygin, Larisa Prokhvatilova, Iliya Chvala

**Affiliations:** Federal Center for Animal Health, 600901 Vladimir, Russia

**Keywords:** avian influenza, genetic reassortment, nomadic birds, Caspian Sea

## Abstract

In 2021, several isolates of the H5N5 avian influenza virus (AIV) were detected in Europe and the Russian Federation, which differed from those detected in 2020. Genetic analysis revealed a relationship between the highly pathogenic avian influenza H5N5 subtype, detected in Europe, and some isolates detected in the Russian Federation territory in 2020–2021: it was shown that both originated in the Caspian Sea regions around the autumn of 2020. The appearance of H5N5 subtype viruses in the spring of 2021 in Europe and the Russian Federation was not associated with the mass migration of birds from Africa. The results of the analysis revealed the presence of a deletion in the stem of a neuraminidase between bp 139 and 204 (open reading frame). It has been shown that AIVs of the H5N5 subtype are capable of long-term circulation in wild bird populations with the possibility of reassortment. The results also highlighted the need for careful monitoring of the circulation of AIVs in the Caspian Sea region, the role of which, in the preservation and emergence of new antigenic variants of such viruses in Eurasia, is currently underestimated.

## 1. Introduction

Avian influenza is a highly dangerous viral disease that causes huge economic damage to poultry farming. The highly pathogenic avian influenza (HPAI) H5N1 virus, isolated from domestic geese on a farm in Guangdong Province (China), became epizootic in 1996: since then, highly virulent H5 avian influenza viruses (AIVs) with different neuraminidase subtypes have spread worldwide. After the identification of three isolates, i.e., A/breeder duck/Korea/Gochang1/2014 (H5N8), A/duck/Korea/Buan2/2014 (H5N8), and A/Baikal Teal/Korea/Donglim3/2014 (H5N8), in Korea in 2014, the H5N8 subtype virus spread globally, first to the United States and then to other Asian and European countries [1,2,3]. One of the first H5N8 viruses isolated in the Russian Federation was A/wigeon/Sakha/1/2014, which belonged to the Buan-like genetic lineage [4]; however, the H5N8 AIV belonging to the Gochang-like genetic lineage has been the most widespread since 2016. At the end of 2016 (November–December), the HPAI epizootic situation in the Russian Federation deteriorated, with H5N8 outbreaks being recorded in poultry farms in the Astrakhan and Rostov Oblasts, the Krasnodar Krai, and in the Republic of Kalmykia. In 2017, more than 30 cases of highly virulent H5N8 AIV were detected in poultry flocks in eight regions. Influenza outbreaks caused severe economic losses in poultry industries in the Astrakhan, Rostov, and Moscow Oblasts, and in the Republic of Tatarstan [5]. In 2017–2018, the H5N2 subtype was isolated in the Kostroma Oblast of the Russian Federation, and the H5N5 subtype was detected in the same region in 2020. The origin of both viral subtypes is closely related to the process of gene reassortment of the highly pathogenic H5N8 virus. The emergence in the Russian Federation of new antigenic variants that had not been previously recorded indicates active reassortment with other virus subtypes. A previous study showed the existence of HPAI virus reassortment during the extensive epizootics of 2016–2017 [6]. At the time, seven main groups were identified and designated as follows: CABAD8AA; CAEAF8AA; AAAAA8AA; AADAA8AA; DCBAE5AA; BABAB8AA; and BDBAB8AA [6]. Mine et al. described the allocation of five genotypes within the G2 cluster of the 2.3.4.4. genetic clade [7]. Despite numerous studies being conducted on new AIVs, many questions regarding their origin remain unanswered. In general, researchers currently study the evolution of influenza viruses using one main approach, which consists of the analysis of a large amount of data, to determine the approximate evolutionary direction of viruses: however, viruses other than the general pool may be excluded from the analysis; therefore, even in large research projects, it is not possible to establish the key geographic regions in which new AIV variants originate [6,8]. Based on the migration patterns of wild birds, and phylogenetic analyses reported in Xiong et al., it can be speculated that H5N8 viruses may be spread by wild birds from different geographic regions to Siberia in summer, and that these viruses are spread from Siberia to Central China by migratory birds along the East Asian–Australasian Flyway in autumn [9]. The present study analyzed a small number of new isolates of the H5N5 virus, detected in Russian Federation territory in 2021: the aim was to determine the geographical region where the viruses originated, and to evaluate the occurrence of similar events in the past in this region.

## 2. Materials and Methods

### 2.1. Virus Isolation

A total of 6418 samples of biological material from domestic and wild birds, obtained from 75 regions of the Russian Federation, was examined by PCR, including 4710 samples of internal organs, 1362 samples of feces, 282 cloacal swabs, and 64 tracheal swabs. The viruses were isolated from 10-day-old specific pathogen-free chicken embryos (SPF-CE). A 10–20% suspension was prepared, in a phosphate-buffered solution (pH 7.2–7.4), from the biological material (tracheal and cloacal swabs, internal organs, feces), and was inoculated into the allantoic cavity of the SPF-CE, at 0.2 mL. Extraembryonic fluid was collected from the embryos that died after 24 h of incubation, to conduct further studies.

### 2.2. Identification of the Isolated Viruses

The isolates were identified based on hemagglutinin (HA) and neuraminidase (NA), using the hemagglutination inhibition test, with H1–H16 AIV sera (IZSVe, Padova, Italy) as reference, and the neuraminidase activity inhibition assay, with N1–N9 AIV sera (IZSVe, Padova, Italy) as reference, based on the World Organisation for Animal Health (WOAH) recommendations, and generally accepted methods [10,11].

### 2.3. Reverse Transcription and Real-Time Polymerase Chain Reaction (RT-qPCR)

The total RNA was isolated using “RIBO-sorb” kit (Interlabservice, Moscow, Russia, cat. no. K2-1-Et-100) according to the manufacturer’s instructions. After extracting the total RNA, RT-qPCR was performed in one stage, using the OneStep RT-PCR Kit (Qiagen, Hilden, Germany) and primers for the amplification of the MP, HA, and NA genes of the H5 subtype AIV. The reaction mixture contained 1× RT-PCR buffer, 1.25 mM MgCl_2_, 0.4 mM dNTPs, 0.4 µM forward and reverse primers, and a mixture of reverse transcriptase and polymerase enzymes. A total of 5 µL of RNA was added to 20 µL of reaction mixture. The reverse transcription step was performed for 30 min at 50 °C. The qPCR was started at 95 °C for 10 min (polymerase activation), followed by 40 cycles at 95 °C for 10 s, 55 °C for 35 s, and 72 °C for 10 s.

### 2.4. Conventional Reverse Transcription and Polymerase Chain Reaction (RT-PCR)

RT-PCR was performed in one stage, using the OneStep RT-PCR Kit (Qiagen, Cat. No. 210212) and primers for the amplification of the HA gene of the H5 subtype AIV. The reaction mixture contained 1× RT-PCR buffer, 1.25 mM MgCl_2_, 0.4 mM dNTPs, 0.4 µM forward and reverse primers and a mixture of reverse transcriptase and polymerase enzymes. A total of 5 µL of RNA was added to 20 µL of reaction mixture. The reverse transcription step was performed at 50 °C for 30 min. The PCR was started at 95 °C for 10 min (polymerase activation), followed by 40 cycles at 94 °C for 30 s, 58 °C for 60 s, and 68 °C for 2 min.

### 2.5. Sequencing

The nucleotide sequences of the HA gene fragment were determined using an ABI Prism 3130 automatic sequencer and the BigDye Terminator Cycle Sequencing kit (Applied Biosystems, Waltham, MA, USA) according to the manufacturer’s instructions. Genome-wide sequencing was performed using a MiSeq genetic analyzer (Illumina, Hayward, CA, USA) according to the manufacturer’s instructions (for the resequencing of small genomes). The commercial Nextera XT Library Prep Kit (Illumina, Hayward, CA, USA) and Nextera XT Index Kits (Illumina, Hayward, CA, USA) were used to prepare the libraries. The viral genome was built in GS Reference Mapper v2.7 and GS De Novo Assembler v2.7, and the genome sequencing results were deposited in the GenBank database (Table 1).

### 2.6. Nucleotide Sequences

Nucleotide sequences of the H5 subtype AIV strains were downloaded from the publicly available EpiFlu platform (https://www.gisaid.org/) (accessed on 2 November 2022). The names of avian influenza viruses on the phylogenetic tree are in «Isolate name|Type|Segment|Collection date|Isolate ID» format. We selected 50 maximally similar isolates for each analyzed segment (excluding NA), for viruses A/swan/Rostov/2299-2/2020, A/mute_swan/Romania/11981-1_21VIR3163-5/2021, A/chicken/Senegal/21VIR1084-4/2021, and A/goose/Omsk/01171/2020. Nucleotide sequences of viruses of the H5N1 subtype found in Asia in the period 01.01.2019-30.08.2021 were added for phylogenetic analysis. For the analysis of the neuraminidase gene, a set of 500 maximally similar segments was used. In addition, a number of viruses of other subtypes found in Europe, but close to viruses found from the Russian Federation for individual genes, were used for phylogenetic analysis. Duplicate sequences and sequences with a large number of gaps and uncertainties were removed from the resulting data set.

### 2.7. Analysis of Nucleotide and Corresponding Amino Acid Sequences

Full-size open reading frames (ORFs) were used for analysis, and the sequences were compared in BioEdit v. 7.0.5.3 (https://doi.org/10.3390/v14112321) (accessed on 2 October 2022). The ClustalW multiple alignment program was used to perform the alignment, through which a maximum likelihood phylogenetic tree was generated, using GTR G+I with 200 bootstrap iterations in MEGA 7 [12]. The percentage of trees in which the associated taxa clustered together is indicated next to the branches. The initial tree(s) for the heuristic search were obtained automatically, by applying the Neighbor Join and BioNJ algorithms to a matrix of pairwise distances estimated using the Maximum Composite Likelihood approach, and then by selecting the topology with superior log-likelihood value. To improve clarity, the phylogenetic trees are shown with compressed individual nodes. The full-sized phylogenetic trees are included in the Supplementary Materials.

## 3. Results

**Figure 1:**
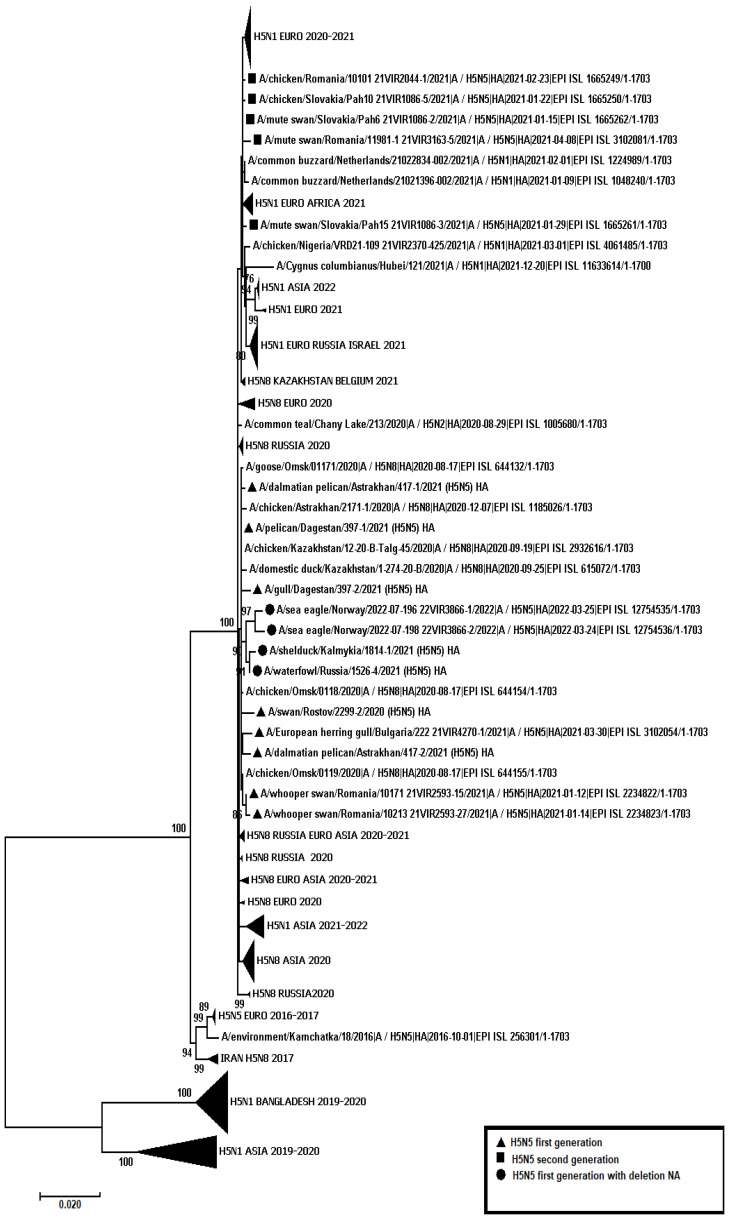
Phylogenetic tree constructed using the Neighbor Join (NJ) method, based on the nucleotide sequence of the HA gene fragment (1–1704 b.p. ORF) of the AIV subtype.

**Figure 2:**
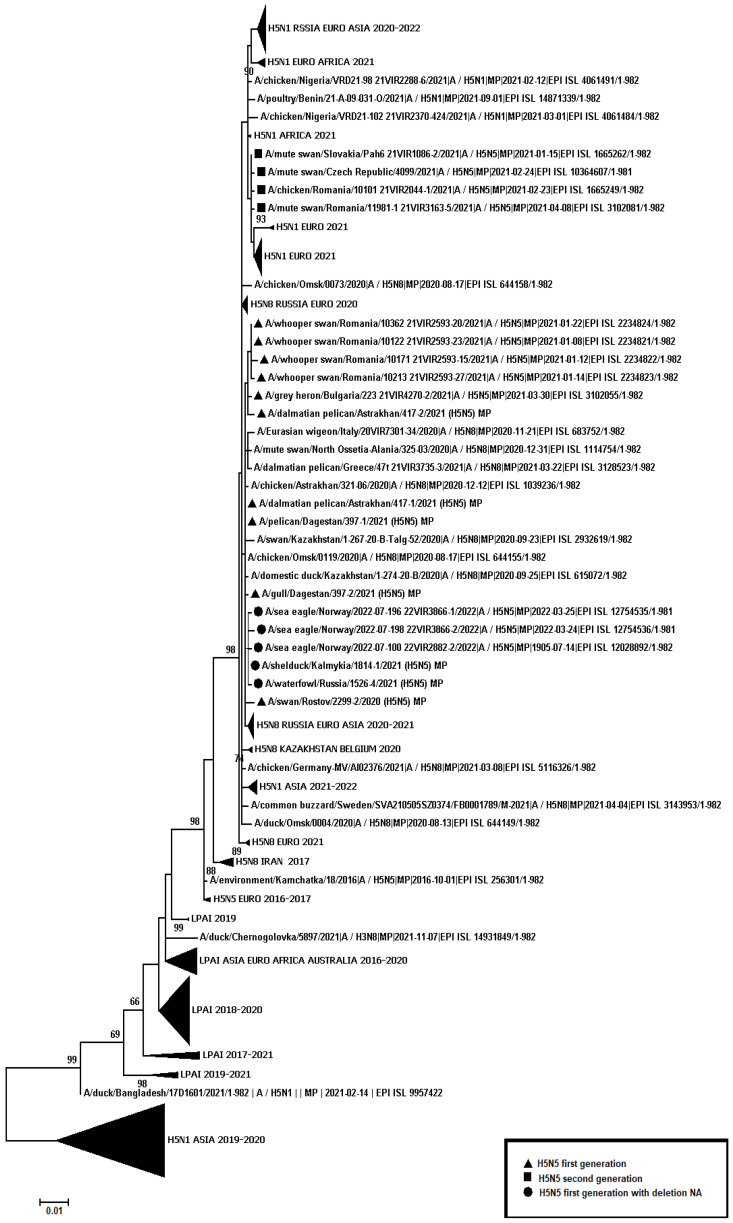
Phylogenetic tree constructed using the NJ method, based on the nucleotide sequence of the MP gene fragment (1–982 b.p. ORFs) of the AIV subtype.

**Figure 3:**
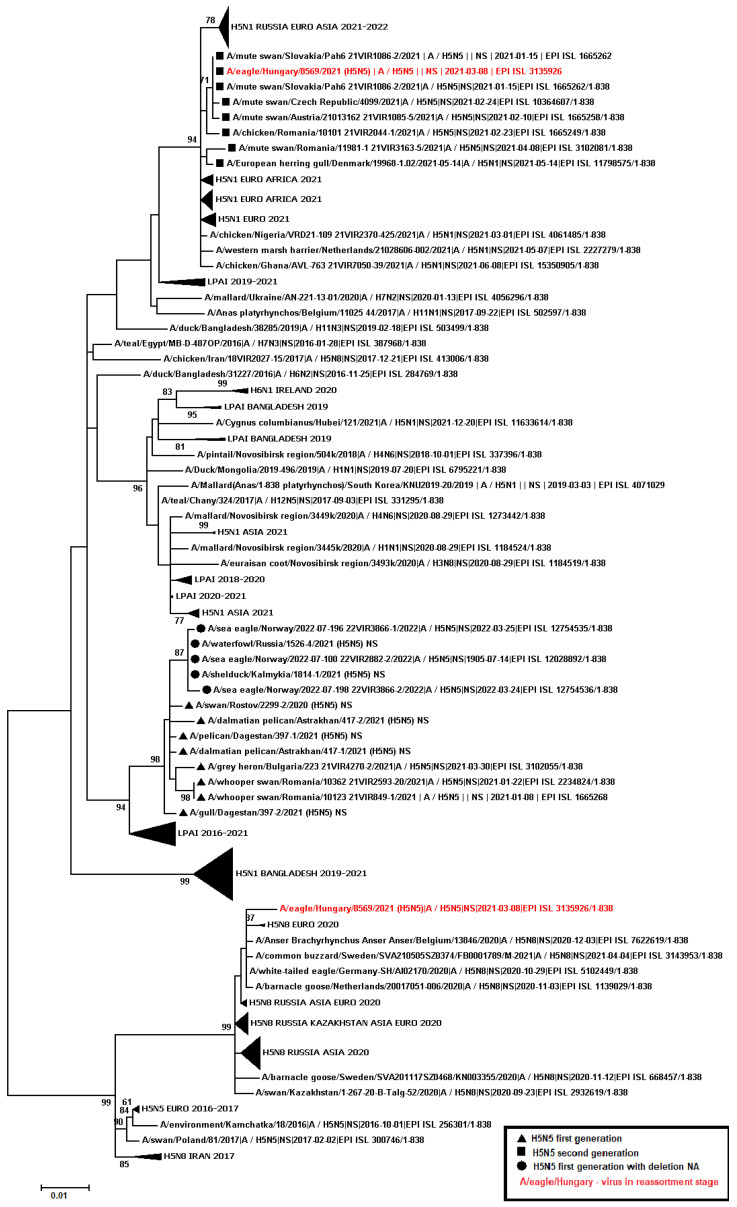
Phylogenetic tree constructed using the NJ method, based on the nucleotide sequence of the NS gene fragment (1–693 bp ORF) of AIV subtype H5.

**Figure 4:**
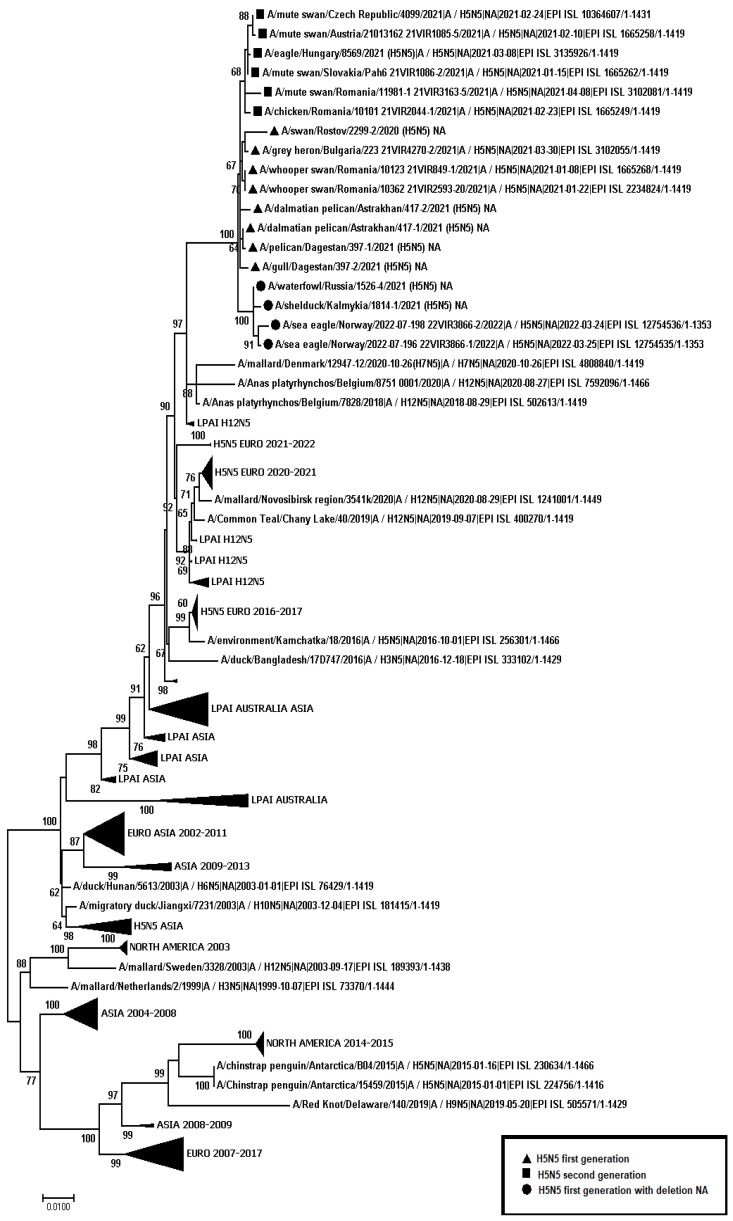
Phylogenetic tree constructed using the NJ method, based on the nucleotide sequence of the NA gene fragment (1–1419 bp ORF) of AIV subtype N5.

**Figure 5:**
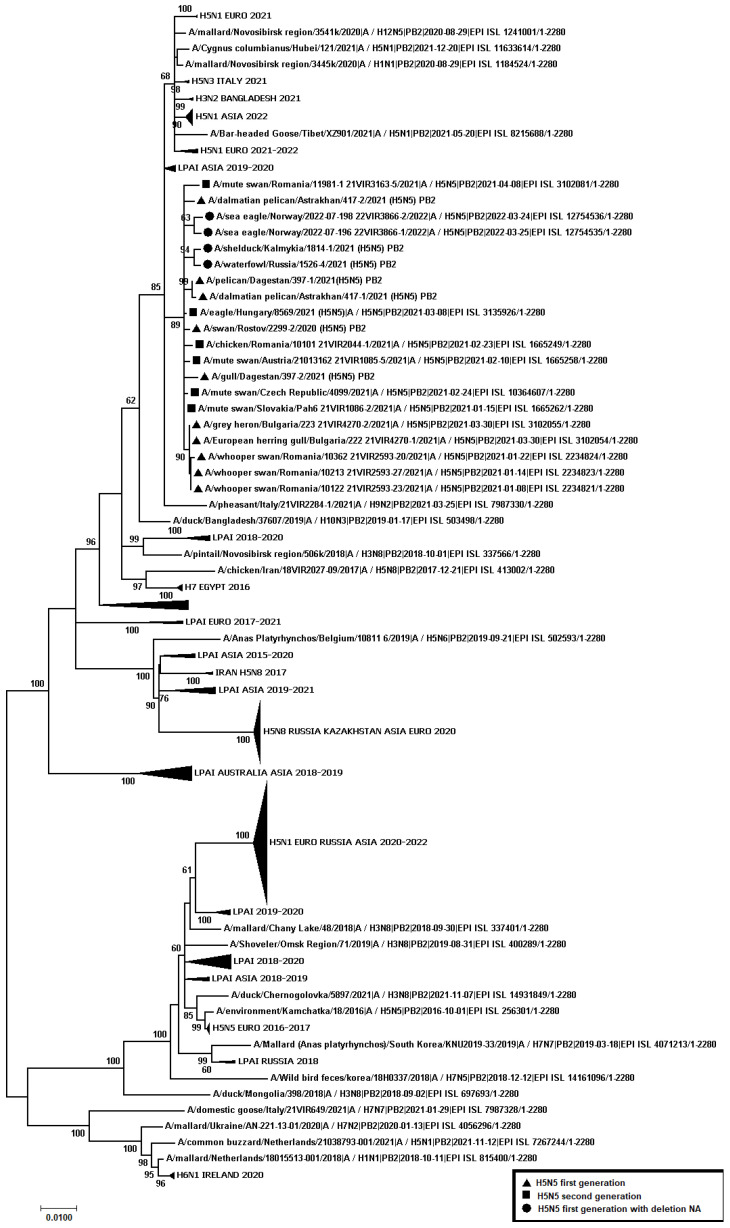
Phylogenetic tree constructed using the NJ method, based on the nucleotide sequence of the PB2 gene fragment (1–2280 bp ORF) of AIV subtype H5.

**Figure 6:**
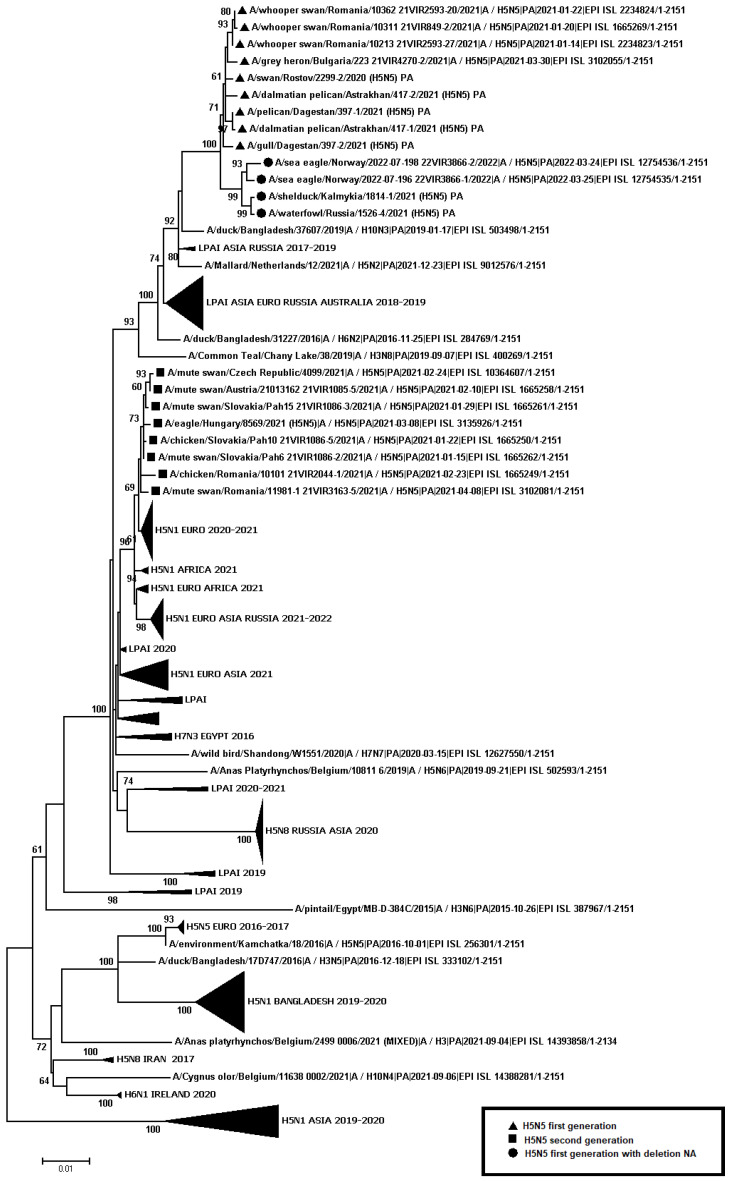
Phylogenetic tree constructed using the NJ method, based on the nucleotide sequence of the PA gene fragment (1–2151 bp ORF) of AIV subtype H5.

**Figure 7:**
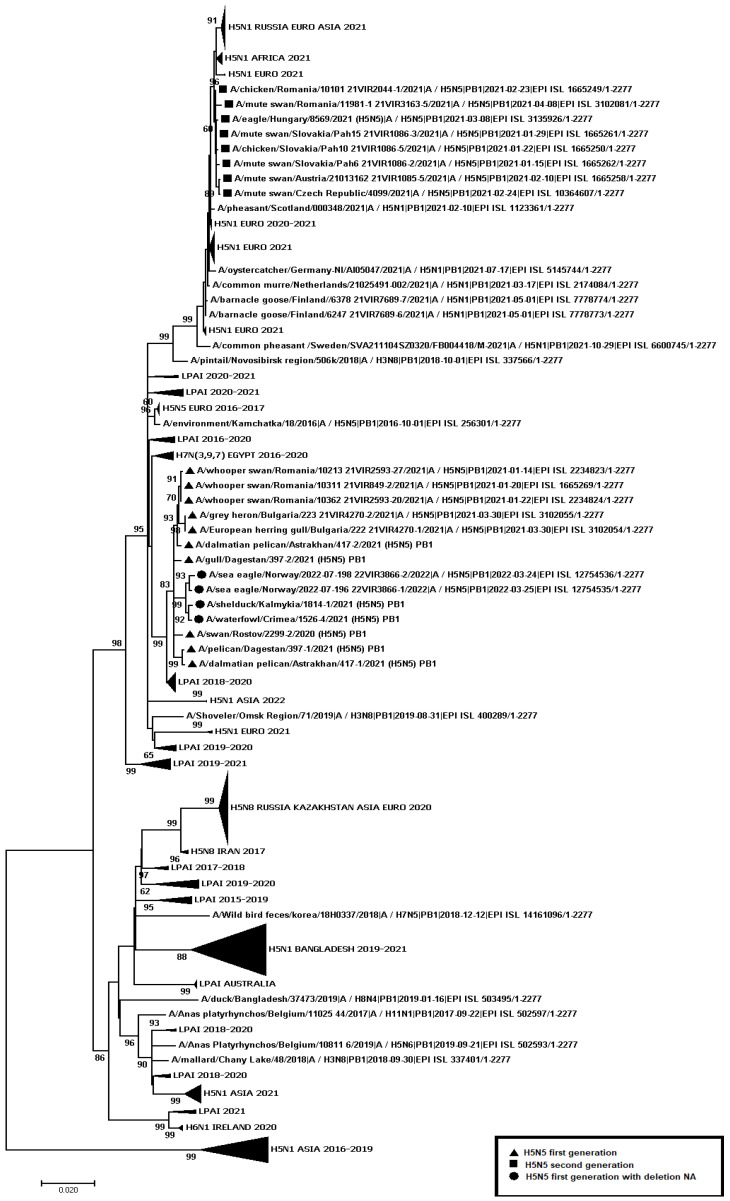
Phylogenetic tree constructed using the NJ method, based on the nucleotide sequence of the PB1 gene fragment (1–2275 bp ORF) of AIV subtype H5.

**Figure 8:**
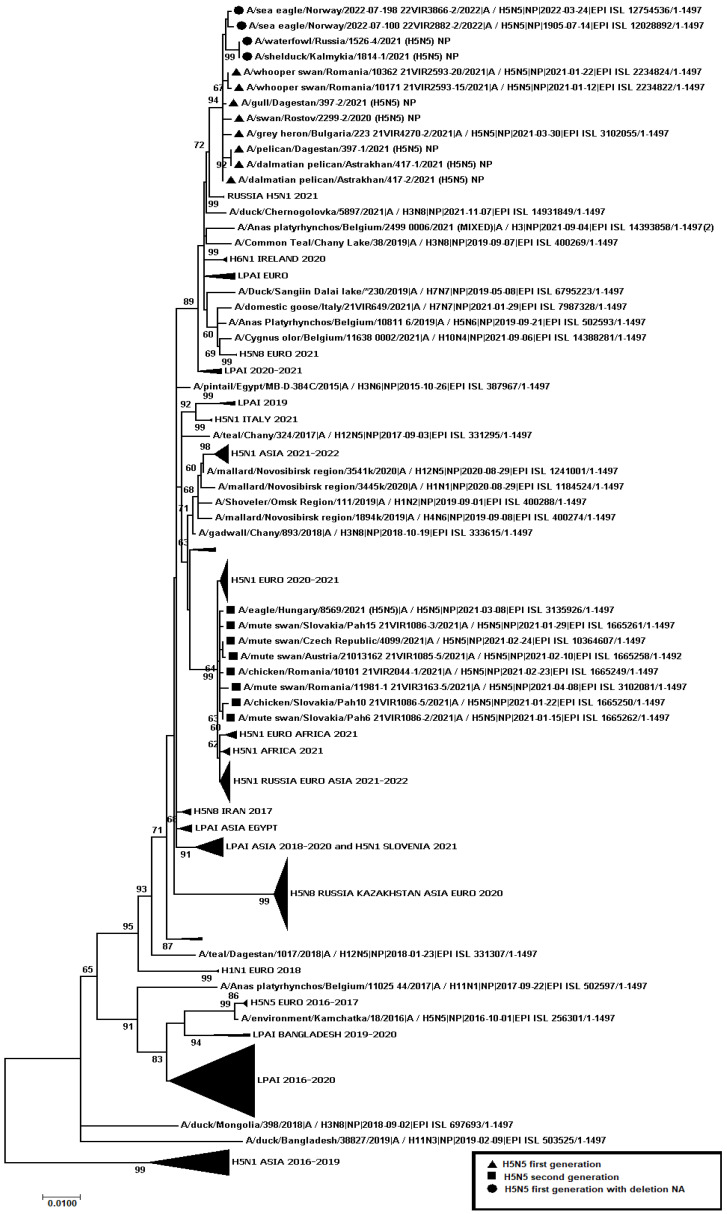
Phylogenetic tree constructed using the NJ method, based on the nucleotide sequence of the NP gene fragment (1–1497 bp ORF) of AIV subtype H5.

A total of 445 samples of biological material from domestic and wild birds, obtained from 75 regions of the Russian Federation, resulted positive to AIVs, specifically to subtypes H5N1(303), H5N8(30), H5N5(18), H4N6(3), H9N2(30), H6N1(2), H3N8(3), and H16N3(4). For 52 samples, it was not possible to establish belonging to subtype by neuraminidase. The FGBI “ARRIAH” investigations, conducted in 2021 and supplemented by the GISAID database, found that, based on the HA gene nucleotide sequence, the HPAIV isolates recovered that year in the Russian Federation belonged to genetic clade 2.3.4.4. (Figure 1). From the point of view of the evolution of AIVs and their transfer to other geographical regions, the following group of H5N5 subtype viruses isolated from the Caspian Sea coast is of particular interest: A/dalmatian pelican/Astrakhan/417-2/2021(H5N5) (recovered on 1 April 2021); A/dalmatian pelican/Astrakhan/417-1/2021(H5N5) (recovered on 1 April 2021); A/pelican/Dagestan/397-1/2021(H5N5) (recovered on 6 April 2021); A/gull/Dagestan/397-2/2021(H5N5) (recovered on 6 April 2021); A/waterfowl/Russia/1526-4/2021(H5N5) (recovered on 28 September 2021); A/shelduck/Kalmykia/1814-1/2021(H5N5) (recovered on 8 November 2021), hereafter collectively referred to as viruses of the Caspian region (VCR). For VCR, a similar clustering of genomic segments was established with the isolates of the H5N5 subtype influenza virus recovered in Europe in winter and spring 2021 (Figure 1, Figure 2, Figure 3, Figure 4, Figure 5, Figure 6, Figure 7 and Figure 8). The phylogenetic analysis of nucleotide sequences of entire viral genomes belonging to the H5N5 subtype suggests that they all evolved through the reassortment of HPAI H5N8 with one unidentified N5 subtype virus. This was confirmed by the dense clustering of viruses carrying the HA and MP genes for H5N5 and HPAI of the H5N8 subtype, previously identified in the Russian Federation (Figure 1 and Figure 2). The exception was the avian influenza virus A/eagle/Hungary/8569/2021. Two NS genes were identified in the A/eagle/Hungary/8569/2021 sample. One NS gene was similar to H5N8 avian influenza viruses. Another gene NS A/eagle/Hungary/8569/2021 was from H5N1 viruses with which reassortment occurred (Figure 3).

The H5N5 subtype isolates recovered in Europe in the autumn of 2020 were also included in the analysis. The phylogenetic analysis showed that, based on genomic segments NA, PA, PB1, PB2, NS, and NP, these isolates had no segments in common with those recovered in 2021 (Figure 3, Figure 4, Figure 5, Figure 6, Figure 7 and Figure 8). In addition to the isolated topology of VCR, it is interesting to note that this group presented the isolate A/swan/Rostov/2299-2/2020 (H5N5), which was identified in the southern Federal District of the Russian Federation on 17 December 2020 (https://wahis.woah.org/#/in-review/3459?reportId=19351&fromPage=event-dashboard-url (accessed on 5 October 2022)). The nucleotide sequence of the NA gene of the A/swan/Rostov/2299-2/2020 isolate was analyzed using the Blastn tool on the GISAID website, and resulted as the first H5N5 influenza virus detected in 2020–2021. The results obtained allowed us to assume that the H5N5 subtype viruses detected in Europe and the Russian Federation in 2021 emerged in the autumn of 2020. Presumably, the H5N5 virus was introduced into Europe at this time via an isolate similar to A/swan/Rostov/2299-2/2020 from the Caspian region. VCR and viruses introduced to Europe (i.e., A/European herring gull/Bulgaria/222 21VIR4270-1/2021, A/grey heron/Bulgaria/223 21VIR4270-2/2021, A/whooper swan/Romania/10123 21VIR849-1/2021, A/whooper swan/Romania/10311 21VIR849-2/2021, A/whooper swan/Romania/10122 21VIR2593-23/2021, A/whooper swan/Romania/10171 21VIR2593-15/2021, A/whooper swan/Romania/10213 21VIR2593-27/2021, and A/whooper swan/Romania/10362 21VIR2593-20/2021) represented the first generation of viruses. In Figure 1, Figure 2, Figure 3, Figure 4, Figure 5, Figure 6, Figure 7 and Figure 8, the first generation of viruses H5N5 subtype are marked by a triangle.

In central Europe, these viruses exchanged genomic segments with H5N1 subtype influenza viruses: this led to the emergence of another group of viruses, which included A/mute_swan/Austria/21013162_21VIR1085-5/2021, A/chicken/Slovakia/Pah10_21VIR1086-5/2021, A/eagle/Hungary/8569/2021, A/mute_swan/Slovakia/Pah15_21VIR1086-3/2021, A/mute_swan/Slovakia/ Pah6_21VIR1086-2/2021, A/chicken/Romania/10101_21VIR2044-1/2021, and A/mute_swan/Romania/11981-1_21VIR3163-5/2021), hereafter referred to as “H5N5 second generation”, and marked by a square in Figure 1, Figure 2, Figure 3, Figure 4, Figure 5, Figure 6, Figure 7 and Figure 8.

In the autumn of 2021, avian influenza viruses of the H5N5 subtype were again detected in the southern Federal District of the Russian Federation: comparative analysis showed that they were very similar to the first avian influenza virus A/swan/Rostov/2299-2/2020 (H5N5). In July 2022, avian influenza viruses of the H5N5 subtype were detected in Norway: they were very similar to the first avian influenza virus A/swan/Rostov/2299-2/2020 (H5N5). Despite viruses A/waterfowl/Russia/1526-4/2021 (H5N5), A/shelduck/Kalmykia/1814-1/2021 (H5N5), A/sea eagle/Norway/2022-07-196 22VIR3866-1/2022, and A/sea eagle/Norway/2022-07-198 22VIR3866-2/2022 being phylogenetically close to the viruses of the first generation, they may have specific biological properties. The listed viruses were originated from viruses of the first generation, as a result of the deletion of the viral neuraminidase: these viruses have a deletion in a viral neuraminidase with a size of 66 bp (139–204 bp ORF). In Figure 1, Figure 2, Figure 3, Figure 4, Figure 5, Figure 6, Figure 7 and Figure 8, viruses with a deletion in the neuraminidase are marked by a circle.

The general scheme of reassortment of genomic segments of the avian influenza virus is presented in Table 2. VCR H5N5 received genes PB1, PB2, PA, NP, NS, and NA from the LPAI HxN5, and genes MP and HA from the HPAI H5N8 influenza virus. In Europe, VCR H5N5 received the genes PB1, PA HA, NP, NS, and MP from avian influenza subtype H5N1.

**Figure 9:**
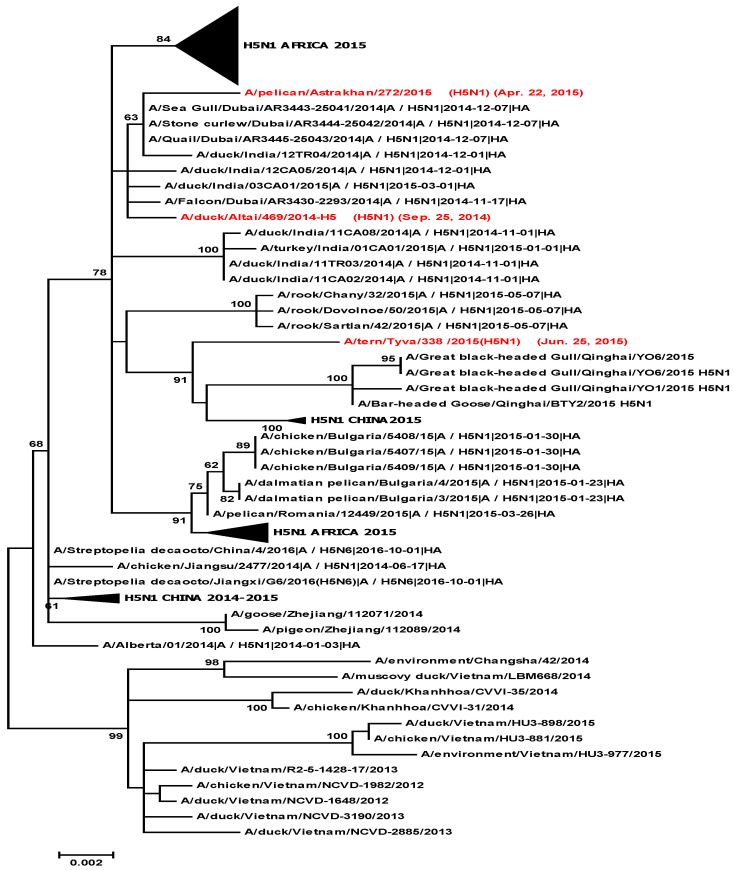
Phylogenetic tree constructed using the NJ method, based on the nucleotide sequence of the HA gene fragment (1–1704 bp ORF) of AIV subtype H5N1. H5N1 avian influenza viruses identified in the Russian Federation in 2014–2015 are marked in red.

We additionally analyzed viruses collected during the 2014–2015 epizootics of HPAI (subtype H5 of genetic clade 2.3.2.1). In 2014, we detected the H5N1 virus A/duck/Altai/469/2014 (H5N1) (recovered on September 25, 2014) in the Altai Republic, Siberia, and closely related viruses were also found in the Middle East and Africa, causing numerous epizootic outbreaks. In 2015, there were no mass outbreaks of HPAIV (subtype H5 of genetic clade 2.3.2.1) in the Russian Federation or Europe. In spring 2015, the introduction of viruses into Europe affected only Bulgaria and Romania. Only a few cases of HPAI were detected in the Russian Federation in spring (A/pelican/Astrakhan/272/2015 in the Astrakhan Oblast (recovered on 22 April 2015)) and in autumn (A/tern/Tyva/338/2015 in the Tyva Republic (recovered on 25 June 2015) and Novosibirsk region; unpublished data). Following phylogenetic analysis, the A/pelican/Astrakhan/272/2015 isolate detected in the Astrakhan Oblast in spring was clustered with isolates of genetic clade 2.3.2.1. recorded in the Russian Federation (in the Altai region) and in the Middle East in autumn 2014. This isolate differed from other viruses of genetic clade 2.3.2.1 detected in Africa in winter 2014–2015 and in Europe in 2015 (Figure 9), indicating that its introduction was not associated with birds migrating from the African continent in spring.

## 4. Discussion

We suppose that a number of outbreaks of avian influenza virus in Russia (H5N5 in 2021, H5N1 in 2015) and Europe (H5N6 in 2017, H5N5 in 2021) were not associated with mass seasonal migration of birds: these outbreaks were caused by the introduction of avian influenza viruses from the insufficiently explored Caspian region, by nomadic birds. The proposed model of “winter circulation” of AIVs, including HPAI in the Caspian region and its transfer via small populations of nomadic birds, can fill in the gaps in the pattern of AIV diffusion across Eurasian territory at the end of the mass migration season. It is possible that the long-term circulation of LPAI and HPAI within bird populations in the Caspian Sea and the Caucasus Mountains allows the AIV to maintain its genetic diversity through antigenic shift and the interspecies transmission associated with it.

The results obtained in this study led to an assumption about the “winter circulation” of influenza viruses in the Caspian region, where numerous wild waterfowl species overwinter. Due to global warming, the ice on the Volga River is formed later now; therefore, the ice-free river delta with its extensive thickets provides an excellent shelter and food resources for wintering birds. When the Volga freezes, the remaining birds can undertake a relatively short migration along the Caspian coast, gathering in other ice-free areas. Such gatherings of different wild avian species around small water bodies may facilitate an antigenic shift among influenza viruses, and also cause interspecies transmission, as new antigenic variants emerge. Comparative analysis of epizootics in 2014–2015 and 2016–2017 showed that the number of bird species affected by AIVs has significantly increased [13].

The lower number of H5N5 subtype viruses compared to that of H5N1 viruses detected in 2021 can be explained by the fact that the former were introduced into Europe via a small bird population. In central Europe, these viruses exchanged genomic segments with H5N1 subtype influenza viruses. As a result, the following group of viruses appeared: A/mute_swan/Austria/21013162_21VIR1085-5/2021, A/chicken/Slovakia/Pah10_21VIR1086-5/2021, A/eagle/Hungary/8569/2021, A/mute_swan/Slovakia/Pah15_21VIR1086-3/2021, A/mute_swan/Slovakia/ Pah6_21VIR1086-2/2021, A/chicken/Romania/10101_21VIR2044-1/2021, and A/mute_swan/Romania/11981-1_21VIR3163-5/2021—the H5N5 second generation. The emergence of viruses with truncated neuraminidase (e.g., A/waterfowl/Crimea/1526-4/2021, A/shelduck/Kalmykia/1814-1/2021, A/sea_eagle/Norway/2022-07-198_22VIR3866-2/2022, and A/sea_eagle/Norway/2022-07-196_22VIR3866-1/2022) and the possible mechanisms behind it deserve special attention. It has been shown that deletions in the stem of neuraminidases appear as a result of the adaptation of viruses from wild waterfowl to poultry [14], and the adaptation of the H5 subtype to poultry is possible only if there is strong immunity against this virus within bird flocks: thus, the occurrence of these adapted viruses may be possible only in free-range breeding farms, where poultry are vaccinated against the H5 subtype AIV or where stable circulation of low pathogenic H5 AIV is observed.

**Figure 10:**
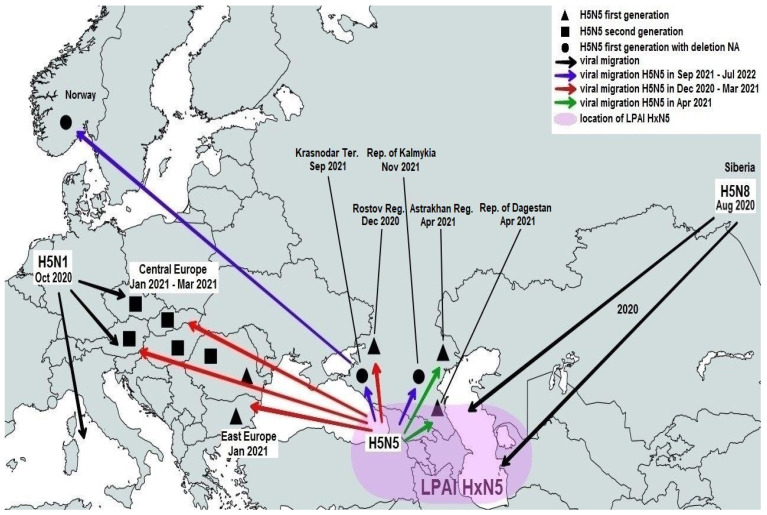
Spread of H5N5 avian influenza virus during 2021–2022.

We assume that in the autumn of 2020, a new H5N5 was formed in the territory of the Caspian region, as a result of the reassortment of avian influenza virus H5N8 and the low pathogenic viruses HxN5. With a small bird population, the avian influenza virus H5N5 migrated to central Europe: there, the virus reassorted with H5N1 again. However, some of the avian influenza virus H5N5 survived in the Caspian region, and in the spring it entered the territory of the Russian Federation. In autumn, with a new bird migration, the mutated avian influenza virus H5N5 again migrated towards Europe and reached Norway, where it was detected in 2022 (Figure 10).

As the secondary reassortants of the H5N5 influenza virus also retained their topology, via genomic segments related to other H5 subtype viruses isolated in 2021, a single reassortment event may be assumed: this may also confirm that the H5N5 virus was introduced into Europe via a small bird population. Until April 2021, VCR maintained the topology of phylogenetic trees based on the genomic segments observed in the A/swan/Rostov/2299-2/2020 ancestor strain. In our opinion, a reverse genetic shift is unlikely in this case, when birds return from Europe to the Caspian coast. The long-term preservation of the same genomic configuration in the H5N5 subtype AIV indicates that it can circulate for an extended period in wild bird populations, with the possibility of reassortment. The detection of VCR in spring 2021, first in Dagestan, and then in the Astrakhan Oblast, may be associated with the migration of some bird populations from the Caucasus Mountains or from Iran. Numerous wetland systems in Iran are important stopover and wintering areas along the African–West Eurasian mass migration routes. This country hosts a large number of migratory species of the Middle East [15]. Most migratory Anatidae species in Iran are found in the northern wetlands and in the southern Caspian lowlands [16].

The role of the Caucasus in preserving the genetic diversity of AIVs has not been sufficiently studied, despite the fact that three migratory routes intersect in this territory: the Central Asian, East African/West Asian, and the Black Sea–Mediterranean [17]. Poen et al. 2019 detected only two H5/HPAIV isolates among 1095 influenza-positive samples [18]. Such an abundance of low pathogenic viruses can be a great source for genetic reassortments and a large serological overlap, and can contribute to the preservation of HPAI in populations. HPAIV isolates recovered in Georgia in 2017–2018 exhibited a genomic structure for which it was difficult to determine the parent viruses (Figure 1, Figure 2, Figure 3, Figure 4 and Figure 5). Some researchers have suggested the possibility that HPAIVs adapt to persist in wild bird populations. Apparently, numerous reassortments occur in bird populations with many low pathogenic influenza viruses, allowing HPAIVs to persist and evolve [17,19,20].

The spread of HPAIVs belonging to genetic clade 2.3.2.1 in autumn 2014 was associated with a deviation from the usual migration route. As a result, viruses that were genetically similar to that detected in the Altai region were identified on November 1, 2014, in India. Based on phylogenetic analysis, the HPAIV isolates detected in India presented a greater affinity with those discovered in the Middle East than with the HPAIV of genetic clade 2.3.2.1 detected in China in 2015. According to the data available, the virus (genetic clade 2.3.2.1) started to spread in Siberia in autumn 2014, went through Central Asia and reached the Middle East, and finally moved further into North Africa. The penetration into India was apparently connected with other migration routes that intersect with the autumn migration routes of birds flying from Siberia to Africa: apparently, nomadic birds (pelicans, swans, peregrine falcons, etc.) play an equally significant role in the transfer and formation of new variants of the influenza virus. The staging area of nomadic birds in the region shows that birds can migrate from west to east, and vice versa. Such migrations allow the transfer of viruses between latitudes, and when the season of mass migration is over. Viruses detected in Siberia in 2015 could have entered the country with nomadic birds flying in from India through the central regions of China. The detection of the A/pelican/Astrakhan/272/2015 isolate in the spring of 2015, and its genetic differences relative to the influenza virus detected in Europe in the same season and year, indicate another source of the introduction of the virus into Russian Federation territory in 2015. The similarity between the A/pelican/Astrakhan/272/2015 isolate and the influenza viruses found in autumn 2014 in the Middle East also points to a similar scenario, in which H5N1 and H5N5 viruses may have been introduced to some extent into the Russian Federation in 2015 and 2021, respectively.

We analyzed European epizootics caused by HPAIV subtype H5N6. Comparative analysis of the first isolates of this subtype, detected in Greece in 2017 (i.e., A/chicken/Greece/39_2017b/2017, etc.), showed that they were reassortants of HPAIV subtype H5N8, which was widely spread in 2016 across Eurasia, and of the low pathogenic isolate A/mallard duck/Georgia/3/2016 (A/H4N6): the first cases of the virus in January–February that year were reported in the closely located regions of Romania and Greece. In regard to A/chicken/Greece/39_2017b/2017, it can be argued that the AIVs detected in the Caucasus Mountains participated in its evolution: thus, we observed a similar introduction into Europe of the H5N6 and H5N5 viruses in 2017 and 2021, respectively. The only recorded case of H5N6 in the Russian Federation in 2018 was not associated with the introduction of AIVs from Europe, as phylogenetic analysis showed that this subtype was related to isolates from East and Southeast Asia. The lack of up-to-date information on AIVs in Turkey and Iran hinders the development of models that can describe the introduction of reassortant H5N5 viruses to the Russian Federation in 2021, and of H5N6 viruses to Europe in 2017.

The intensification of agriculture, its expansion into new lands, and the natural succession of lakes and swamps reduce the suitable wintering areas for migratory birds, and force them to migrate more frequently in winter, in search of more favorable conditions [21]. The reduction of wintering areas in Iran may also force the birds into taking new migration routes, consequently pushing them out into other geographical regions, including Europe.

## Figures and Tables

**Table 1:** Access numbers for the viral genome segments deposited in GenBank.

Name of the Virus	Access Number
A/dalmatian pelican/Astrakhan/417-1/2021 (H5N5)	OP597554–OP597561
A/dalmatian pelican/Astrakhan/417-2/2021(H5N5)	OP597562–OP597569
A/pelican/Dagestan/397-1/2021(H5N5)	OP597570–OP597577
A/gull/Dagestan/397-2/2021(H5N5)	OP597578–OP597585
A/swan/Rostov/2299-2/2020 (H5N5)	OP597586–OP597593
A/shelduck/Kalmykia/1814-1/2021 (H5N5)	OP597594–OP597601
A/waterfowl/Russia/1526-4/2021 (H5N5)	OP597602–OP597609
A/pelican/Tumen/932-1/2021 (H5N1)	OP597634–OP597641
A/pelican/Tumen/1027-1/2021 (H5N1)	OP597626–OP597633
A/common teal/Chelyabinsk/1379-1/2021 (H5N1)	OP597610–OP597617
A/goose/Chelyabinsk/1341-3/2021	OP597618–OP597625
A/duck/Altai/469/2014 (H5N1)	OP597551
A/pelican/Astrakhan/272/2015 (H5N1)	OP597552
A/tern/Tyva/338/2015 (H5N1)	OP597553

**Table 2:** Reassortment of genomic segments of subtype H5N5 avian influenza viruses identified in Europe and Russia in 2021.

	LPAI HxN5	HPAI H5N8	HPAI H5N5 First Generation	HPAI H5N1
A/swan/Rostov/2299-2/2020 first generation	PB1, PB2, PA, NP, NS, NA	MP, HA		
A/eagle/Hungary/8569/2021 virus in reassortment stage	PB1, PB2, PA, NP, NA	MP, HA, NS		NS
A/mute_swan/Slovakia/ Pah6_21VIR1086-2/2021 second generation			NA, PB2	MP, HA, PB1, PA, NP, NS

## Data Availability

Data release will occur after the article is published. The article shows the numbers under which the data was deposited in GenBank.

## Supplementary Materials

The following supporting information can be downloaded at: https://www.mdpi.com/article/10.3390/v14122725/s1.
