# Supplementary material for: Analysis of Avian Influenza (H5N5) Viruses Isolated in the Southwestern European Part of the Russian Federation in 2020–2021
Source: Viruses. 2022 Dec 6;14(12):2725. doi: 10.3390/v14122725 (PMC9783257; doi:10.3390/v14122725)
Supplement: Supplementary file 1 [file viruses-14-02725-s001.zip › Short instruction.pdf]

Download the software and install it. We used version 7.0. However, files may be opened in a version 10.0.

https://www.megasoftware.net/ or read (https://www.ncbi.nlm.nih.gov/pmc/articles/PMC8210823/)

In program version 7.0. To view short branches, you can use the option “Display Only Topology” (1).

Select the branch you are interested in (2).

Select an option “Show Subtree Separately” (3). The contents of the selected branch will be displayed in a new window.

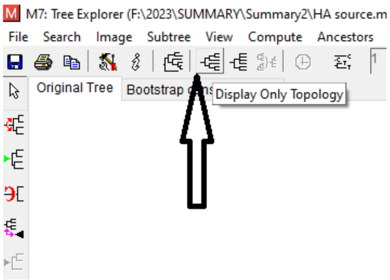

1

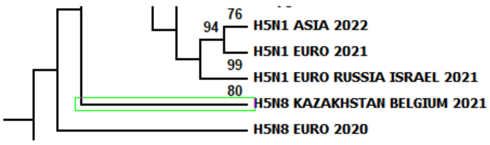

2

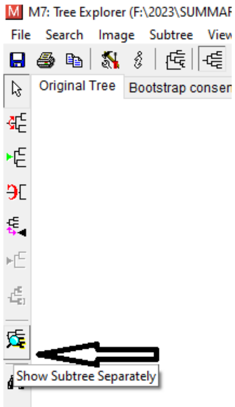

3
